# The dog prostate cancer (DPC-1) model: a reliable tool for molecular imaging of prostate tumors and metastases

**DOI:** 10.1186/s13550-015-0155-6

**Published:** 2015-12-30

**Authors:** Simone Chevalier, Serge Moffett, Eric Turcotte, Murillo Luz, Lyne Chauvette, Vilma Derbekyan, Eleonora Scarlata, Fatima Zouanat, Armen G. Aprikian, Maurice Anidjar

**Affiliations:** Urologic Oncology Research Group, Division of Urology, Department of Surgery, McGill University, and Research Institute (RI) of McGill University Health Centre (MUHC), 1001 Boulevard Décarie, Montréal, QC H4A 3J1 Canada; ProScan, Rx Pharma Inc., 5800 Royalmount Avenue, Montréal, QC H4P 1K5 Canada; Department of Nuclear Medicine, Centre Hospitalier Universitaire de Sherbrooke, 580, rue Bowen Sud, Sherbrooke, QC J1G 2E8 Canada; Department of Nuclear Medicine, McGill University and MUHC, 1001 Boulevard Décarie, Montréal, QC H4A 3J1 Canada; Department of Urology, Jewish General Hospital, 3755 Chemin de la Côte-Sainte-Catherine, Montreal, QC H3T 1E2 Canada

**Keywords:** Prostate cancer, Dog prostate, PSMA, SPECT/CT imaging

## Abstract

**Background:**

Clinical applicability of newly discovered reagents for molecular imaging is hampered by the lack of translational models. As the dog prostate cancer (DPC-1) model recapitulates in dogs the natural history of prostate cancer in man, we tested the feasibility of single-photon emission computed tomography (SPECT)/CT imaging in this model using an anti-prostate-specific membrane antigen (PSMA)/17G1 antibody as the radiotracer.

**Methods:**

Immunoblots and immunohistochemistry (IHC) with 17G1 were performed on canine and human prostate cancer cell lines and tissues. Five dogs with DPC-1 tumors were enrolled for pelvic and, in some instances, thoracic SPECT/CT procedures, also repeated over time. Controls included ^111^indium (In)-17G1 prior to DPC-1 implantation and ^111^In-immunoglobulins (IgGs) prior to imaging with ^111^In-17G1 in dogs bearing prostatic DPC-1 tumors.

**Results:**

17G1 cross-reactivity with canine PSMA (and J591) was confirmed by protein analyses on DPC-1, LNCaP, and PC-3 cell lines and IHC of dog vs. human prostate tissue sections. 17G1 stained luminal cells and DPC-1 cancer cells in dog prostates similarly to human luminal and cancer cells of patients and LNCaP xenografts. SPECT/CT imaging revealed low uptake (≤2.1) of both ^111^In-17G1 in normal dog prostates and ^111^In-IgGs in growing DPC-1 prostate tumors comparatively to ^111^In-17G1 uptake of 3.6 increasing up to 6.5 values in prostate with DPC-1 lesions. Images showed a diffused pattern and, occasionally, a peripheral doughnut-shape-like pattern. Numerous sacro-iliac lymph nodes and lung lesions were detected with contrast ratios of 5.2 and 3.0, respectively. The highest values were observed in pelvic bones (11 and 19) of two dogs, next confirmed as PSMA-positive metastases.

**Conclusions:**

This proof-of-concept PSMA-based SPECT/CT molecular imaging detecting primary prostate tumors and metastases in canines with high cancer burden speaks in favor of this large model’s utility to facilitate technology transfer to the clinic and accelerate applications of new tools and modalities for tumor staging in patients.

**Electronic supplementary material:**

The online version of this article (doi:10.1186/s13550-015-0155-6) contains supplementary material, which is available to authorized users.

## Background

Prostate cancer (PCa) is the second most common cause of cancer in men and among the leading causes of death by cancer worldwide [1]. Modalities developed to assess risk categories and select best therapeutic options comprise digital rectal examination, levels of circulating prostate-specific antigen (PSA), and Gleason score of prostate biopsies (referred to as the D’Amico criteria) [2, 3], also coupled to percentage of positive biopsies and patient age (known as the UCSF-CAPRA score) [4]. Local staging may be improved by multiparametric magnetic resonance imaging (MRI) [5], although sensitivity to detect lymph node (LN) metastases remains low (~40 %) [6]. Whole body diffusion MRI appears accurate for detection of bone metastases [7], even if non-specific and relying on differential vascularization and density of benign vs. cancer tissues [5].

Molecular imaging with radiotracers by single-photon emission computed tomography (SPECT) and positron emission tomography (PET) has opened a new era in cancer staging by incorporating tumor cell biology into the equation [8]. The discovery of prostate-specific membrane antigen (PSMA) overexpression in PCa [9–11] has paved the way to targeted rather than functional imaging, as achieved by PET/CT with ^18^F-choline uptake in proliferating PCa cells [12]. Among PSMA-attractive features are prostate specificity for luminal secretory cells with minimal expression in other tissues and increasing levels from low grade to metastatic castrate-resistant prostate cancer (CRPC) [9, 10].

Historically, PSMA-targeted molecular imaging was achieved through ^111^indium (In)-labeled monoclonal antibodies (mAbs) like 7E11 (first generation) and J591 (second generation) which permitted the detection of necrotic tissue vs. LN and bone metastases due to the recognition of intracellular vs. extracellular epitopes, respectively [9, 10]. Recently, PET/CT with ^89^Zr-labeled J591 has identified prostatic tumor foci in 8 of 11 (72 %) patients prior to prostatectomy, although the standardized uptake value did not correlate significantly with the Gleason score [13]. To counter the slow elimination of full-length mAbs from circulation and also optimize tumor contrast, PSMA derivatives were developed, notably radiolabeled minibodies (diabody/J591 mAb), small molecule inhibitors (MIP1072, MIP1095), and ligands [10, 14–19]. Utility in clinical practice is being demonstrated; for instance, PET/MRI with ^68^Ga-based PSMA ligands revealed LN and bone metastases in patients with progressive disease after conventional treatments [16]. The inhibitor MIP1095 showed utility for PET/CT target imaging once labeled with ^124^I (iodine) and also tumor therapy in CRPC patients once labeled with ^131^I [19]. These observations highlight molecular imaging as a promising avenue to better stage and treat patients with locally advanced and recurrent PCa.

A major limitation for testing new modalities is the lack of suitable translational models. Of relevance is the unique propensity of the canine species, unlike most mammals, to spontaneously develop PCa with advancing age, although to a lesser frequency than human [20]. Interestingly, PCa in canines mimics the advanced form of the human disease characterized by a high tumor burden and the presence of numerous local and distant metastases, notably to the skeleton [20]. Our team has developed [21] and optimized [22] an orthotopic dog prostate carcinoma (DPC-1) model that recapitulates the advanced form of PCa in dogs. In the present investigation, we obtained proof of concept of the reliability of this model for molecular imaging by SPECT/CT using an ^111^In-labeled mouse mAb (17G1) targeting human PSMA [23].

## Methods

### Antibody production

The generation of anti-PSMA mAbs or 17G1 was detailed elsewhere [23]. Briefly, 17G1 is a murine IgG1k monoclonal antibody obtained by immunizing BALB/c mice with a peptide (^490^Nt-GKSLYESWTKK) derived from the human PSMA extracellular amino acid sequence conjugated to keyhole limpet hemocyanin. The antibody was purified from the supernatant of hybridoma cell culture media by affinity chromatography using a HiTrap protein G column, as per manufacturer’s instructions (GE Healthcare Life Sciences, PA, USA), concentrated to ~1–5 mg proteins per milliliter by ultrafiltration (Millipore, ON, Canada) and stored at −20 °C in 10 % glycerol. Purity was ascertained by Coomassie staining of proteins separated by sodium dodecyl sulfate polyacrylamide gel electrophoresis (SDS-PAGE). The 17G1 specificity for human PSMA was reported [23] with detection of the recombinant protein and a ~100-kDa band in Western blots of PSMA-positive LNCaP but not PSMA-negative (control) PC-3 [24]. The J591 mAb was similarly purified from the hybridoma obtained from the American Type Culture Collection (ATCC, MD, USA) and used for comparison purposes.

### Prostate cancer cell lines

Cell lines, canine DPC-1 (patent US2003013191: Cussenot and Villette, France), and human PC-3 (CRL-143) and LNCaP (CRL-1740) (ATCC) were maintained in RPMI 1640 containing 10 % fetal bovine serum and 1 % antibiotic/antimycotic solution (Invitrogen Life Technologies, Inc., ON, Canada).

### Dot and Western blots and silver staining

Cells in monolayers were washed twice with ice-cold phosphate-buffered saline (PBS), recovered by scraping, and collected by low-speed centrifugation (1000×*g*, 10 min) prior to protein solubilization in RIPA buffer (20 mM Tris-HCl pH 7.4, 150 mM NaCl, 1 % Nonidet P-40, 1 mM Na vanadate, 10 μg/ml aprotinin, 10 μg/ml leupeptin). Lysates were clarified by centrifugation (14,000×*g*, 5 min) and supernatants recovered to determine protein concentrations (BCA assays), using bovine serum albumin (BSA) as the standard.

For dot blots, protein samples (1–4 μg) were directly applied to nitrocellulose membranes and incubated in 5 % milk blocking solution in 1 % Tween in Tris-base buffer saline (TTBS) for 60 min and then with 17G1 and J591 (dilution 1:1000) in PBS for 2 h at room temperature. Membranes were washed three times with 5 % milk TTBS, incubated for 60 min with secondary mouse antibodies conjugated to peroxidase (1:3000; Thermo Scientific, Il, USA), and washed again with TTBS to reveal reactivity with the enhanced chemiluminescence (ECL) reagent (Denville Scientific, NJ, USA).

For Western blotting, proteins (20 and 40 μg) were separated by SDS-PAGE gel (4–15 % acrylamide gradient) and transferred onto nitrocellulose membranes (as above for dot blots) for incubation with 17G1 and J591 (1:500 in milk TTBS, overnight at 4 °C). PSMA was also immunoprecipitated (750 μg proteins) with 17G1 (2 μg) overnight at 4 °C. Antigen:antibody complexes were recovered by sepharose-G beads, washed three times with PBS, and separated by SDS-PAGE. At this point, proteins were either directly stained in gels with silver nitrate, as per manufacturer’s instructions (Pierce® Silver Stain, Thermo Scientific), or transferred to membranes for Western blotting with 17G1 as above.

### Immunofluorescence (IF) and immunohistochemistry (IHC)

Cells cultured on poly-L-Lysine-coated eight-well plastic chambers were fixed with 3.7 % paraformaldehyde; preincubated (5 min) with 50 mmol/l NH_4_Cl (10 min), 0.5 % Triton X-100 in PBS, and 0.5 % BSA-PBS solution (10 min), prior overnight incubation with 17G1 (1:250 in BSA); and revealed by mouse secondary antibodies, coupled to rhodamine (CY3; Invitrogen Life Technologies, Inc.), as described in [25]. Nuclei were counterstained with 4,6-diamidino-2-phenylindole (DAPI) (Life Technologies Inc., Burlington, Canada) and analyzed by microscopy (Olympus IX81).

For immunohistochemistry (IHC), sections (4 μm) from formaldehyde-fixed paraffin-embedded blocks of LNCaP s.c. xenografts grown in mice [26], dog prostates, DPC-1 tumors, or other dog-harvested tissues [22], as well as archival human prostate specimens, were stained with 17G1, using the EnVision kit (DAKO, ON, Canada). Unless indicated, incubations were performed at room temperature. Steps included are as follows: rehydration with graded alcohol, permeabilization with 1 % Triton X-100/PBS (30 min), antigen retrieval with 0.01 M sodium citrate buffer, pH 6.0 (15 min, 95 °C), quenching of endogenous peroxidase activity with 3 % H_2_O_2_ (30 min), and non-specific staining with a blocking reagent (30 min; Millipore). After three washes (5 min, PBS), sections were next incubated overnight at 4 °C with 17G1 (1:200) in PBS containing 1 % blocking solution. After three more washes, biotinylated secondary Abs (goat anti-mouse IgGs) and streptavidin-peroxidase conjugate were added to amplify the signal. The reaction was revealed within 1 min after the addition of chromogen, 0.006 % 3,3′-diaminobenzidine tetrahydrochloride. Sections were counterstained with hematoxylin, dehydrated, and mounted. Negative controls included sections incubated without 17G1. The staining was assessed by two independent observers blind to the identity of the sections. A consensus was reached on positive or negative signals and cellular/tissue distribution. Images were acquired using an Olympus microscope (IX81).

### Implantation of DPC-1 cells

Intact mongrel dogs (*n* = 5; Laka, Montréal, QC, Canada) of different breeds, body sizes (25–38 kg), and age (≥5 years old) were individually housed within animal facilities certified for Good Animal Practice care by the Canadian Council on Animal Care. They were fed a regular canine diet (except the night before surgery) and had free access to water. Conditions to implant DPC-1 cells within the prostate and follow tumor development over time were as depicted earlier [22]. Prostate biopsies (transabdominal ultrasound-guided approach) were positive by ~6–8 weeks.

### Conjugation and radiolabeling of 17G1

All solutions were prepared with deionized water (Thermo Fisher Scientific) and treated with a Chelex-100 resin, prewashed in 0.5 M sodium acetate pH 6.3 (Bio-Rad; Laboratories; CA, USA) to remove traces of metal ions. To covalently conjugate 17G1 (or mouse IgGs) to 1,4,7,10-tetraazacyclododecane-1,4,7,10-tetraacetic acid (DOTA), the mAb buffer was replaced by 0.1 M sodium carbonate, pH 9.0, by overnight dialysis (Spectrum Laboratories; CA, USA) or centrifugal ultrafiltration (Millipore) at 4 °C. The 17G1 solution (1–5 mg proteins per milliliter) was mixed with 50 molar excess of DOTA-NHS-Ester (Macrocyclics, TX, USA) solubilized in dimethylformamide at 35 mg/ml and incubated for 30 min (at room temperature). The 17G1-DOTA conjugate was immediately separated from unconjugated DOTA-NHS-Ester and other chemicals by repeated centrifugal ultrafiltration and a final wash with 0.3 M ammonium acetate, pH 5.0. The protein concentration of conjugated 17G1 was measured with the Pierce Coomassie Plus kit (Thermo Fisher Scientific).

The number of DOTA molecules linked to 17G1 was determined by MALDI-TOFF mass spectrometry analysis. A mass shift of 2436 Da (compared to unconjugated Ab) confirmed the successful addition of DOTA (646.4 Da), at ~3.7 chelate molecules per 17G1 molecule. At this 17G1:chelate ratio, the 17G1-DOTA reactivity was comparable to 17G1 alone, as measured by ELISA.

Radiolabeling of 17G1 (or IgGs)-DOTA conjugates was carried out no more than 2 days before injection. Briefly, the 17G1-DOTA was mixed with 1–4 mCi of ^111^In-Chloride (Nordion, ON, Canada) in 0.01 M HCl per milligram of mAb and incubated at 43 °C for 60 min. The radiolabeled mAb was washed repeatedly (three times) with PBS by centrifugal ultrafiltration to remove unincorporated radionuclide. The protein concentration was measured (Bradford assays).

Radiochemical purity was determined using 3 μl of ^111^In-DOTA-17G1 (or ^111^In-DOTA-mAb-IgGs), which was incubated for 15 min with an equivalent volume of 1 % diethylene triamine pentaacetic acid (DTPA), pH 5.5, to chelate residual free ^111^In. An aliquot was submitted to thin layer chromatography (10-cm silica-gel-impregnated glass fiber strip; TLC-SG, Agilent; CA, USA) developed in 1 % DTPA, pH 5.5. When the solvent front reached the top, the strip was cut to count the radioactivity (gamma counter) of 17G1-DOTA at the origin and unbound ^111^In at the solvent front.

### SPECT/CT imaging

Procedures were carried out in each animal (*n* = 5) as a function of enrolment (time 0) with follow-up in months (Additional file 1: Figure S1). At the time of injection, the specific ^111^In-17G1 (or control ^111^In-IgGs) radioactivity ranged from 0.9 to 4.5 mCi/mg (mean of 2.6 mCi/mg) with less than 2 % free ^111^In. Most dogs had more than one SPECT/CT session with ^111^In-DOTA-17G1 (several weeks apart). Two animals had additional imaging with ^111^In-DOTA-IgGs.

The ^111^In-DOTA-17G1 (or radiolabeled IgGs) was injected in bolus through an i.v. saline line in a front leg at a total mean dose of 0.037 mg mAbs (0.023–0.104 mg) per kilogram. Unless otherwise indicated, SPECT/CT was performed 2 days later. Pelvic and, in some instances, thoracic imaging were carried out in the same session. The bladder was flushed with saline using a urinary catheter before image acquisition and maintained during procedures to minimize bladder uptake and obtain clearer images of the lower urinary tract. Beside areas of interest, the radiotracer was seen in the liver, heart, and salivary glands in agreement with the literature [9]. Subsequent urine analysis by gel filtration revealed only free ^111^In. Experimental end points ranged from 2 to 5 months.

### Pharmacokinetics

Blood was sampled in anticoagulant tubes to perform 17G1 pharmacokinetics prior to and over time post-injection (5, 15, 30 min and daily up to 10 days). Specimens were kept at 4 °C during sampling and next centrifuged to measure plasmatic 17G1 using a mouse IgG ELISA kit (Roche Life Science, IN, USA).

### Image analysis

Images were obtained with an Infinia Hawkeye4 Hybrid GE camera. SPECT/CT data were analyzed with a clinical workstation (Segami OASIS, Segami Corp, USA). Images were reconstructed using OSEM3D algorithm with CT attenuation correction (hybrid reconstruction, 4 iterations, 10 subsets). Lesion location, size, and signal (S) to background (B) ratio (S/B) were noted for every lesion judged abnormal either on CT or SPECT. Scans were read chronologically for all animals. Readers were blinded to the presence of tumors and injected radiotracers (^111^In-17G1 vs. ^111^In-IgGs) at different time points.

### Histopathology

Euthanasia (90 mg pentobarbital per kilogram i.v.) was induced under anesthesia 1 week after the last SPECT/CT imaging. The abdominal, pelvic, and thoracic cavities were inspected by the veterinary pathologist (ES). The prostate, bladder, and adjacent tissues like the iliosacral lymph center (medial iliac, internal iliac, and sacral LNs) were collected en bloc as well as lungs with visible metastases and suspicious pelvic bone segments. The prostate was weighed, measured, and inked prior to fixation in 10 % buffered formaldehyde. In some instances, lung metastases of varying size were dissected on ice, weighed, and counted to assess remaining radioactivity. The fixed prostate was cut from the cranial to caudal (3–5-mm-thick slices with central urethra) for paraffin-embedding. Whole-mounted sectioning (4 μm) of blocks allowed an overall appreciation of DPC-1 tumor foci once stained with hematoxylin and eosin (H&E) and expression of PSMA through IHC with 17G1, as described above. Other tissues were similarly processed. The time of fixation was extended to 48 h for bone segments, next decalcified in 5 % nitric acid until softness, and paraffin-embedded [22].

## Results

### 17G1 cross-reacts with canine PSMA

**Fig. 1:**
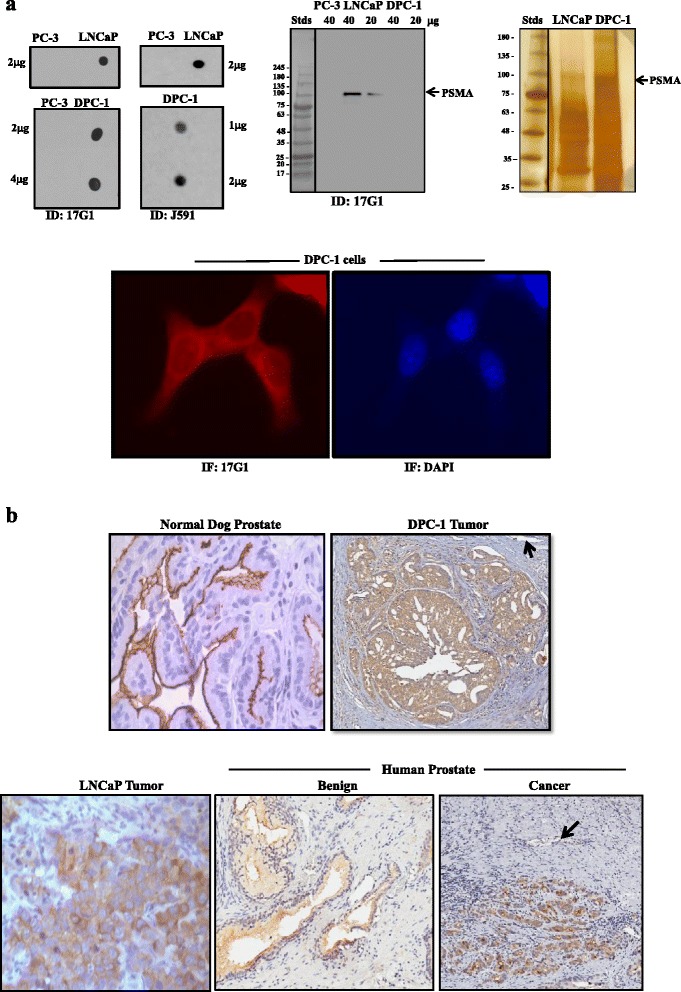
17G1 cross-reactivity with canine PSMA. **a** Protein extracts of PCa cell lines, human (PC-3 and LNCaP) and canine (DPC-1), probed with 17G1 and J591 in dot (*top left*) and Western (*top middle*) blots and also submitted to immunoprecipitation of PSMA in DPC-1 and LNCaP protein extracts and SDS-PAGE gels to stain proteins with silver nitrate (*top right*). *Arrow* indicates the PSMA position at ~100 kDa. PSMA expression in DPC-1 cells, as detected by immunofluorescence (IF) with 17G1 (*red staining*; *lower left*) and DAPI nuclear staining (in *blue*; *lower right*). **b** PSMA expression by IHC with 17G1 in the canine prostate (*top left*) and primary DPC-1 tumor (*top right*), human LNCaP tumor xenograft (*lower left*), benign human prostate (*lower middle*), and PCa tissue (*lower right*). *Arrow* indicates unstained endothelial cells

PSMA protein sequences in human and canine are highly homologous [27], notably in the amino acid sequence 490–500 of the human peptide (GKSLYESW*TK*K) used to generate 17G1 [23] compared to the equivalent canine amino acid sequence (GKSLYESW*NE*K). Accordingly, the reactivity of 17G1 towards canine PSMA was ascertained and compared to J591. Dot blots (Fig. 1a) illustrate a strong signal of both mAbs on DPC-1 and LNCaP (positive control) and no reactivity on PC-3 proteins (negative control) [23, 24] (despite increasing quantities). No signal was detected using mouse IgGs (not shown). In Western blots, 17G1 detected a ~100-kDa band in LNCaP cell extracts but no band in DPC-1 and PC-3, in agreement with earlier reports [23, 24]. Similarly, Western blots with J591 yielded a positive signal only in LNCaP (data not shown).

The possibility that 17G1 recognizes the native PSMA protein in DPC-1 cells was explored by immunoprecipitation with 17G1, followed by SDS-PAGE gels and silver staining of proteins. Results confirmed the presence of a silver-stained ~100-kDa band in both LNCaP and DPC-1 (Fig. 1a), whereas reprobing immunoprecipitated PSMA with 17G1 only led to a signal at ~100 kDa in LNCaP (data not shown). Altogether, these findings support the recognition of a conformational epitope by 17G1 in the native canine PSMA protein. PSMA expression in DPC-1 cells was also confirmed by immunofluorescence (Fig. 1a) while there was no signal with control IgGs (data not shown).

In IHC (Fig. 1b), 17G1 was highly specific for the epithelium, staining luminal cells in dog, and human benign prostates and cancer cells in prostate tumors from patients, DPC-1 prostate tumor in dogs, and LNCaP xenografts. The 17G1-PSMA reactivity was particularly concentrated at the apical membrane of the secretory epithelium of the canine prostate. Yet, some cytoplasmic reactivity was occasionally observed. The signal was cytoplasmic in luminal cells of the human benign prostate although the apical pole may occasionally be stained. A comparable signal was detected in prostate tumor cells, human (patients and LNCaP) and canine (DPC-1-derived).

Endothelial cells of blood vessels were negative in tissues examined, both canine (normal and DPC-1 tumors) and human (benign and cancer) prostates (Fig. 1b).

### SPECT/CT imaging with ^111^In-17G1 detects prostatic tumors

**Fig. 2:**
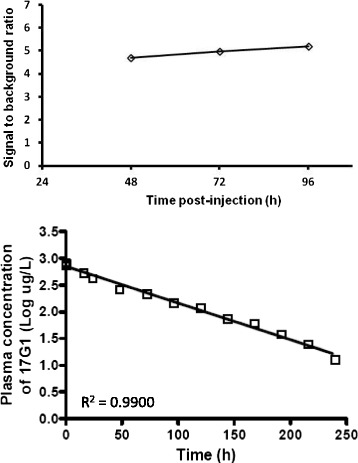
Timing for 17G1 imaging and pharmacokinetics. The radiotracer (^111^In-17G1) was injected as described in the “Methods” section. A first SPECT/CT imaging of the prostate was performed after 48 h and then daily for two more days (*top panel*). Lesion-to-background ratio was determined and plotted (*top panel*). Blood was sampled from prior to and over time post-injection till 10 days and processed to determine the plasma concentration of 17G1 as mentioned in the “Methods” section, plotted as the logarithmic 17G1 concentration vs. time (lower panel) and fitted to a straight line (*R* ^2^ = 0.99) to calculate the half-life elimination

The optimal time for image acquisition of ^111^In-17G1 in the prostate was first assessed. The determination of the S/B ratio over time (Fig. 2, top panel), with no significant improvement at 72 or 96 h compared to 48 h; the half-life of circulating 17G1 of 43.8 h, a time period indicating a rapid elimination and significantly reduced bioavailability at the latest time points (Fig. 2, lower panel); and the 2.8-day ^111^In half-life decay justified the choice of the 48-h time point as being optimal for further imaging.

**Fig. 3:**
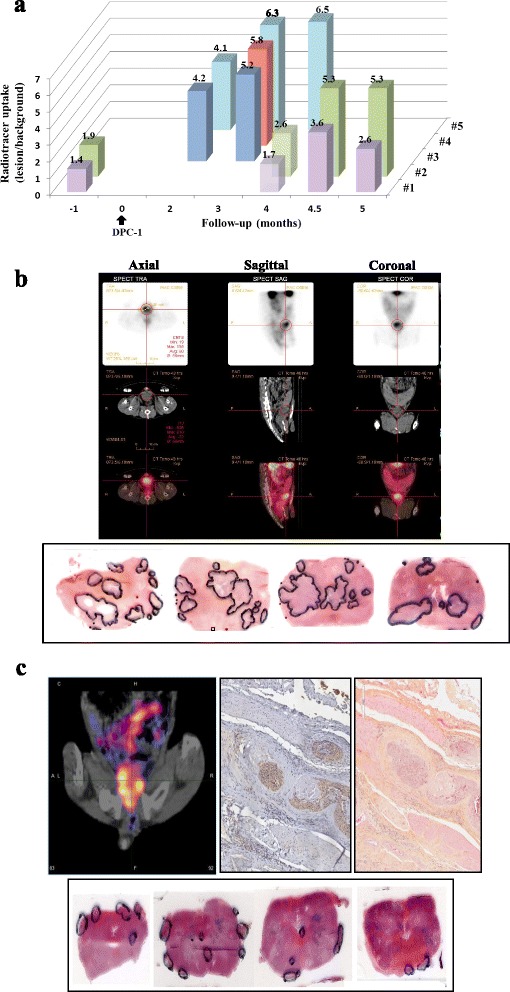
Specificity of prostate imaging with ^111^In-17G1. **a** Tracer uptake in the prostate. Dogs #1 and #2 served as controls to establish a threshold for significance. ^111^In-17G1 was injected prior to DPC-1 cells implantation and at 4.5 months of tumor growth (biopsy-proven between 6 and 8 weeks) along with ^111^In-mouse IgGs at 4 months. **b** Representative SPECT/CT axial, sagittal, and coronal images of ^111^In-17G1 uptake (*top*) in a large portion of the prostate of dog #5 and corresponding tumor foci mapped in sections from consecutive whole-mounted blocks (*lower*). **c** Example of SPECT/CT image with ^111^In-17G1 uptake in the periphery of the prostate (*left top*), confirmed by mapping DPC-1 tumor foci at the capsule (*lower panel*), PSMA expression by IHC (*middle top*) and corresponding H&E staining (*right top*) ×20

Specificity was ascertained in two dogs (see Additional file 1: Figure S1, experimental procedures). Negative controls included are as follows: first, the ^111^In-17G1 antibody uptake in the prostate prior to DPC-1 cell implantation, which was minimal (Fig. 3a), with a mean S/B ratio of 1.65 (dog #1, 1.4; dog #2, 1.9). Second, in the same dogs with biopsy-proven intra-prostatic DPC-1 tumors, the mean S/B of ^111^In-mouse IgGs in the prostate determined at 4 months post-implantation was 2.1 (dog #1, 1.7; dog #2, 2.6) (Fig. 3a). The S/B threshold for significance was then arbitrarily fixed at 3.0, a ratio which can be visually perceptible as positive in clinical practice. The subsequent injection of ^111^In-17G1 nearly 2 weeks later (Fig. 3a) yielded a mean prostate S/B of 4.5 (dog #1, 3.6; dog #2, 5.3). The maximal 17G1 radiotracer uptake for the five dogs was 6.5 (Fig. 3a), and for four dogs imaged more than once, the specific uptake increased or remained the same over time (except dog #1), a finding in support of tumor growth.

Representative SPECT/CT fusion images of the ^111^In-17G1 uptake in the prostate (Fig. 3b) revealed a diffused signal localized within the anterior two third region of the gland. Serial whole-mount prostate sections confirmed the presence of several tumor foci at histopathology, which could account for the observed tracer uptake pattern (Fig. 3b). In one instance (dog #1), the radiotracer uptake displayed a doughnut-shaped more peripheral pattern (Fig. 3c), which was mirrored histopathologically by tumor foci reaching the capsule and beyond. Indeed, after 4–5 months post-DPC-1 implantation, extraprostatic extension was a common feature to all dogs (not shown). Finally, PSMA expression in DPC-1 tumors was confirmed by IHC with 17G1. An example of tumor foci at the periphery of the prostate with diffused cytoplasmic staining is shown (Fig. 3c). With the prostate weight varying from 8 to 47 g, the tumor load may represent a significant volume in this model.

### SPECT/CT imaging with ^111^In-17G1 detects LN metastases

**Fig. 4:**
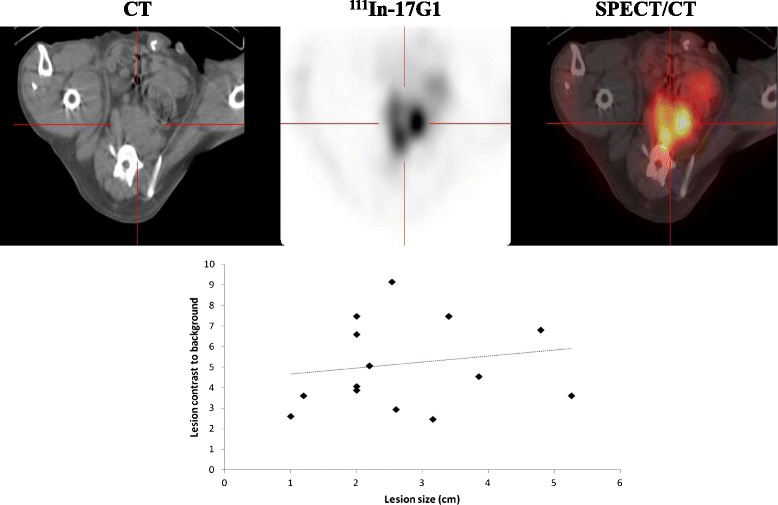
SPECT/CT imaging with ^111^In-17G1 reveals lesions in LNs. Pelvic scan showing sacro-iliac LNs by CT (*left*) with radiotracer uptake (*middle*) and fusion SPECT/CT image (*right*). The radiotracer to background uptake measured in several LNs weakly correlated with size (*lower panel*)

Several foci of tracer uptake in the pelvic area of dogs harboring DPC-1 tumors (Fig. 4) were observed in fusion images and next ascribed to enlarged sacro-iliac LN metastases detectable with 17G1 in each dog at the first imaging session, the earliest being at 2 months post-implantation. A representative example of imaged LN lesions in dog #1 at ~19 weeks is included (Fig. 4); the radiotracer uptake varied slightly with LN size although the correlation was very weak (*R*^2^ = 0.03) and not significant. The uptake even decreased over time when LNs became larger and necrotic.

The mean S/B ratio in LNs was 5.2 ± 2.1, and the average size of detected LN lesions was 2.7 ± 1.2 cm. The smallest LN was of ~1 cm, with a S/B ratio of 2.5. At necropsy, enlarged sacro-iliac LNs of varying sizes were collected, the largest ones being often necrotic in line with imaging results. In IHC, DPC-1 tumor cells in LNs were 17G1-positive with a diffused cytoplasmic signal (Additional file 2: Figure S2). Some reactivity was also observed in the cell debris of necrotic LNs.

### SPECT/CT imaging of DPC-1 lung nodules with ^111^In-17G1

**Fig. 5:**
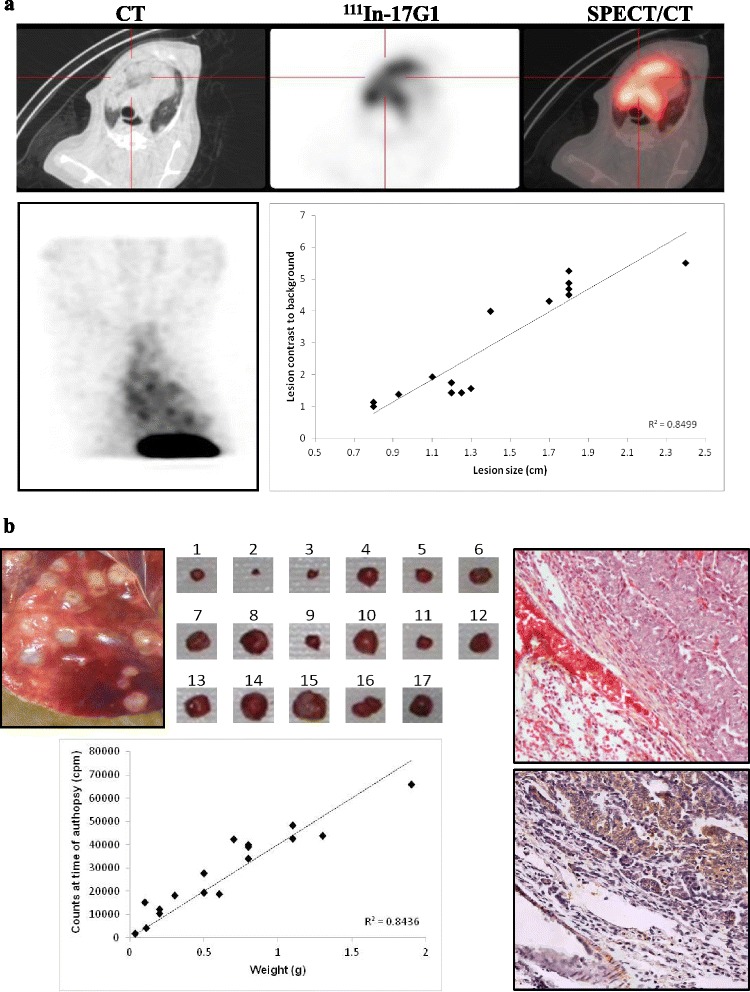
SPECT/CT imaging with ^111^In-17G1 reveals lung metastases. **a** Thoracic scan showing DPC-1 lesions in lungs (*top*): CT (*left*) and radiotracer uptake (*middle*) with fusion image (*right*). Numerous small lesions were visible with the 17G1 radiotracer by SPECT (*lower left*). Lesion contrast to background plotted as a function of nodule size (*lower right*). **b** Lung metastases visible (*top left*) at necropsy performed 1 week after the last SPECT/CT imaging session. Subset of metastases were dissected (*top middle*) and weighed to count remaining radioactivity; the radiotracer uptake correlates with weight of metastases (*lower left*), in line with SPECT/CT imaging data. DPC-1 cells in lung metastases (H&E, *top right*) express high PSMA levels (IHC, *lower right*) (×40).

Considering the high incidence rate of prostate-derived lung nodules in dogs with spontaneous prostate cancer [28, 29] and the DPC-1 model [22], thoracic SPECT/CT imaging was performed in three dogs (#3–5). A representative fusion image revealing the radiotracer uptake in several lesions is shown (Fig. 5a). The specific radiotracer uptake correlated significantly with the size of pulmonary nodules (*R*^2^ = 0.85), with an average S/B ratio of 3.0 ± 1.7 (1–5.5) (Fig. 5, top middle; dog #1 at 24 weeks). The smallest SPECT/CT detected lung nodules measured 1.4 ± 0.5 cm (0.8–2.4 cm), although smaller lesions were anatomically visible on CT (Fig. 5a).

**Fig. 6:**
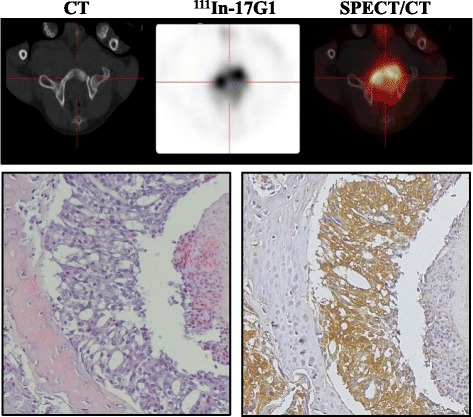
SPECT/CT imaging with ^111^In-17G1 reveals bone metastases. View of pelvic bone scan by CT (*top left*), radiotracer uptake (*top middle*), and fused SPECT/CT (*top right*). DPC-1 cells in bone metastases (H&E, *lower left*) and 17G1 IHC staining for PSMA (*lower right*) (×40)

Pulmonary nodules excised 5 days after the last imaging session still contained significant residual radiotracer (^111^In-) (Fig. 5b), which correlated linearly with their size (*R*^2^ = 0.84); counts ranged from 1700 cpm for a 30-mg nodule to 65,800 cpm for a 1.9-g nodule. The abundance of lung nodules (greater than 200) set the tumor volume at a very high level. DPC-1 cells of lung metastases strongly expressed PSMA (Fig. 5b).

### SPECT/CT imaging with ^111^In-17G1 detects DPC-1 bone metastases

Bone metastases are a common feature of spontaneous PCa in dogs [28–30] and also observed in the DPC-1 model [22]. SPECT/CT pelvic images were further analyzed for the presence of ^111^In-17G1 tracer in bone segments (Fig. 6). A remarkably high uptake was detected in an obturator bone of dog #3 and iliac bone of dog #5 at 4 months post-DPC-1 cell implantation, with S/B ratios of 11.6 and 19.0, respectively. DPC-1 cells in metastatic bones were confirmed to strongly express PSMA (Fig. 6).

## Discussion

This investigation is the first of its kind demonstrating that prostate tumors and associated metastases can be successfully detected by molecular imaging in the orthotopic DPC-1 model recapitulating in large dogs the advanced form of human PCa [21, 22]. PSMA was a reasonable target to choose in this proof-of-concept study given its reported expression in DPC-1 tumors [21] and confirmed in this study. We selected the 17G1 mAb [23] for imaging as it was raised against a human PSMA peptide highly homologous to the corresponding peptide sequence in canine PSMA [23, 27]. Moreover and similarly to J591, 17G1 recognizes the human PSMA protein, recombinant and as a ~100-kDa band in LNCaP. Consistently, cross-reactivity and specificity of 17G1 with canine PSMA were confirmed by different means: (1) dot blots with DPC-1 and LNCaP but not PC-3 proteins yielded a strong reactivity, in agreement with the literature [31]; (2) 17G1 immunoprecipitated a DPC-1 and LNCaP silver-stained band migrating at ~100 kDa in SDS-PAGE gels; and finally, (3) a similar band was detected by 17G1 in Western blots of LNCaP proteins but not DPC-1, thus strongly supporting the recognition of the native protein in both cell lines along with the denatured protein in LNCaP. Yet, PSMA was reported to be a ~100–110-kDa glycoprotein in LNCaP, consisting of a 84-kDa protein moiety predicted from the cDNA and carbohydrates accounting for 20–25 % of its mass [32]. The reported homology between the human and canine protein sequence (82 % identity) [27] together with our findings of a similar protein band in LNCaP and DPC-1 suggest comparable glycosylation patterns. Nonetheless, the fact that 17G1 recognized native forms of PSMA is most valuable in the context of molecular imaging whereby intravenously injected radiotracers recognize accessible epitopes in native proteins.

An observation supporting the use of ^111^In-17G1 as a pharmaceutical for SPECT/CT imaging was 17G1’s specificity for adenocarcinoma cells in IHC, with a diffused cytoplasmic and membrane staining pattern in cancer cells of DPC-1, patients’ prostate tumors, and LNCaP xenografts. However, the strong 17G1 staining of PSMA in luminal/secretory cells of the normal dog and human (benign) prostates, as reported in [33], might have been a disadvantage to image intra-prostatic tumors. Yet, the inclusion of controls permitted to discriminate specific from non-specific radiotracer uptake. Despite the slow clearance of mAbs from circulation and tissues [10], a 2-day period between radiotracer injection and imaging was sufficient to minimize non-specific tracer accumulation in most tissues except for the liver and bowel. The first control—^111^In-17G1—injected prior to DPC-1 cell implantation led to a mean radiotracer prostatic accumulation of 1.6 relative to background. The subsequent control—^111^In-radiolabeled IgGs—injected in the same dogs at 4 months from the time of DPC-1 cell implantation (biopsy-proven tumors) led to a mean 2.1 S/B ratio. Based on these values, a threshold of 3.0:1.0 was arbitrarily chosen to define specific tracer uptake, a value visually perceptible as positive in the clinic. Subsequent SPECT/CT sessions (nearly 2-week interval after control IgGs) validated this definition since the ^111^In-17G1 prostatic uptake was then more than twofold higher than controls with a mean prostate-to-background ratio of 4.5. Moreover, sequential imaging sessions in four of the five enrolled dogs indicated increasing prostatic uptake over time to a plateau in three dogs and subsequent decrease in one, likely due to necrosis often seen in large tumor foci. While it seems paradoxical that the elevated PSMA expression (observed in IHC) in the normal canine prostate epithelium did not translate in a high radiotracer uptake, a possible explanation may be a non-accessibility of the antigen, an extracellular epitope of native PSMA, to circulating 17G1 in the normal vs. malignant prostate. For instance, the PSMA protein is particularly concentrated in the apical pole of secretory cells opening onto the acinar lumen of glands. The underlining basal cell layer and basement membrane could represent a barrier separating PSMA-expressing luminal cells from blood vessels in the stroma. Conversely, the loss of glandular architecture and a single cell layer remaining in tumors, together with the loosening of the basement membrane, could allow 17G1 to reach cancer cells. This is supported by the low and high radiotracer prostatic uptake, normal (S/B = 1.6) vs. malignant (S/B up to 6.5), respectively, and organs with proven DPC-1 tumors. As such, the reactivity of an antigen PSMA at the tumor cell surface, with an antibody-circulating radiotracer, implies the accessibility of native molecules to each other. Whether PSMA and 17G1 leak from their respective compartments is not known. Nonetheless, as eluted to, in molecular imaging, 17G1 would bind PSMA in its native conformation.

Our study revealed two SPECT/CT uptake patterns in prostate tumors, one generally diffused and a more peripheral with a doughnut-shaped aspect. Microscopic analyses of prostates were compatible with such patterns, as several tumor foci were dispersed throughout the prostate and beyond the capsule. These findings of molecular imaging were corroborated by the PSMA positivity of tumor cells and metastases, as shown by IHC. Thus, despite the known limited intrinsic resolution of SPECT images (human scanner of medium size animals), PSMA-based prostate imaging might detect extra-capsular extension, a determining factor for the ideal planning of therapeutic approaches. Our data linked the signal intensity to prostate tumor growth over time, found to be best achieved when the extent of tumor necrosis was limited. This paradigm also applied to pelvic LN metastases, which were often necrotic when reaching the experimental end point and as seen in PET scan studies of patients affected by a variety of cancers [34, 35]. SPECT/CT of the dog pelvic area revealed multiple sites of uptake, measuring as low as 1 cm, or 2.7 ± 1.2 cm in average, as ascribed by CT to sacro-iliac lymphadenopathies. The mean radiotracer uptake to background ratio (5.2 ± 2.1) was within the range of values obtained in prostate tumors. However, the signal intensity and size of LN metastases were poorly correlated (*R*^2^ = 0.03). Nonetheless, these findings are encouraging for future optimization given the low sensitivity and specificity of CT or MRI, with a ~40 % detection rate of pelvic LN metastases, based on the classic 1-cm threshold size [6].

Lung metastases are abundant in canines with DPC-1 tumors [21, 22]. As expected, thoracic SPECT/CT imaging with 17G1 revealed numerous small lung lesions averaging 1.4 ± 0.5 cm, with a mean contrast-to-background ratio of 3.0. Smaller lesions (PSMA+ in IHC) were visible on CT but could not be detected when merged with SPECT. This limitation—small lesions not reaching an uptake level high enough to be visible in SPECT—is ascribed to the partial volume effect of the SPECT scanner. Hence, sequential imaging follow-up on dogs having lesions under 1.4 cm demonstrated increasing size with a higher level of tracer uptake, consistent with the partial volume effect. The tracer uptake strongly correlated with the size of imaged lesions, confirmed to be visible metastases (at necropsy) remaining radioactive for several days post-imaging (^111^indium counts correlating with size of metastases) and highly expressing PSMA (IHC). Another remarkable observation was the detection in two animals of pelvic bone metastases (confirmed by histology and expressing PSMA) with a particularly high ^111^In-17G1 uptake (11 and 19 over background), the highest of all imaged DPC-1 tumors despite the reported low permeability of mAbs in solid tumors and notably bone metastases [17].

Overall, this proof-of-concept study is quite unique as it has coupled PSMA-based SPECT/CT images to the growth of DPC-1 prostatic tumors and metastases both local and distant, then histopathologically confirmed and showed to express the targeted PSMA/antigen in a time-wise manner resembling the natural history of prostate cancer. PSMA-based studies are primarily performed on heterotopic xenografts in rodents bearing a relatively low tumor volume [36] compared to canines. This is particularly attractive as, in contrast to most mammalian species, canines spontaneously develop prostate cancer although at a low incidence rate with ageing [20, 29, 37]. Unfortunately, the cancer is generally advanced at diagnosis and has reached the terminal stage [28–30]. The DPC-1 preclinical model would prove to be most advantageous to enhance resolution or track down very small lesions using newly developed radiopharmaceuticals (diabodies, minibodies, small molecule inhibitors), imaging tools (PET/CT, PET/MRI vs. SPECT/CT), etc. It may find substantial utility to test new drugs or therapeutic modalities to destroy a high tumor/metastasis load not achievable in small rodent models. Finally, the use of equipment from the clinical setting in large size animals is attractive and could accelerate applicability of new devices prior moving to patients and impact on disease from time of early diagnosis to late stages.

## Conclusions

This proof-of-concept study confirmed the reliability of the DPC-1 orthotopic model for molecular imaging and recapitulating in large dogs the advanced form of human disease. Tumors are detectable within the prostate and at the capsule along with pelvic lymph nodes and distant lung and osseous metastases. As the tumor load is significant in volume, this model may serve for the development of a wide spectrum of new staging and therapeutic applications.

## Additional files

Additional file 1: Figure S1. Timeline of experimental procedures. Schematic illustration of imaging procedures performed with 17G1 and IgGs from prior to prostatic implantation of DPC-1 cells until end point (‡). Four dogs were submitted to repeated sessions of SPECT/CT imaging as a function of time following DPC-1 cell implantation in the prostate. Additional file 2: Figure S2. PSMA expression in lymph node metastases. Sacro-iliac LNs of varying size harvested at necropsy (top left), with largest ones being often necrotic (lower left). IHC with 17G1 confirmed PSMA expression in DPC-1 cells forming LN metastases (top right) and remaining debris in necrotic ones (lower right) (×40).
