# Supplementary figures and images for: The dog prostate cancer (DPC-1) model: a reliable tool for molecular imaging of prostate tumors and metastases
Source: EJNMMI Res. 2015 Dec 30;5:77. doi: 10.1186/s13550-015-0155-6 (PMC4695479; doi:10.1186/s13550-015-0155-6)

## Slide 1
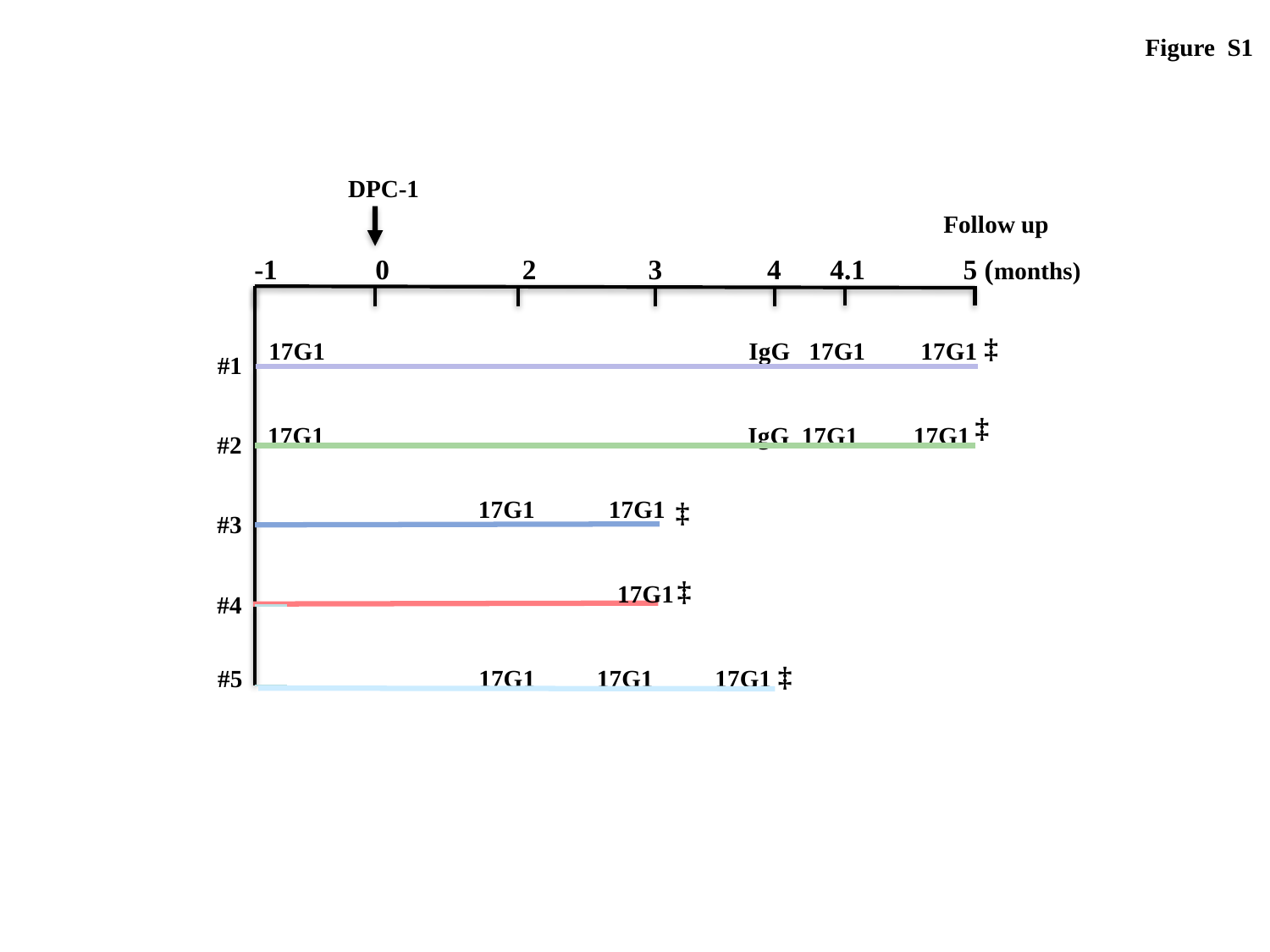

Figure S1
DPC-1
Follow up
-1 0 2 3 4 4.1 5 (months)
 ‡
17G1 IgG 17G1 17G1
#1
‡
 17G1 IgG 17G1 17G1
#2
17G1 17G1
 ‡
#3
 17G1
 ‡
#4
 ‡
17G1 17G1 17G1
#5

Supplement: Additional file 1: Figure S1. — Timeline of experimental procedures. Schematic illustration of imaging procedures performed with 17G1 and IgGs from prior to prostatic implantation of DPC-1 cells until end point (‡). Four dogs were submitted to repeated sessions of SPECT/CT imaging as a function of time following DPC-1 cell implantation in the prostate. [file 13550_2015_155_MOESM1_ESM.pptx]

## Slide 1
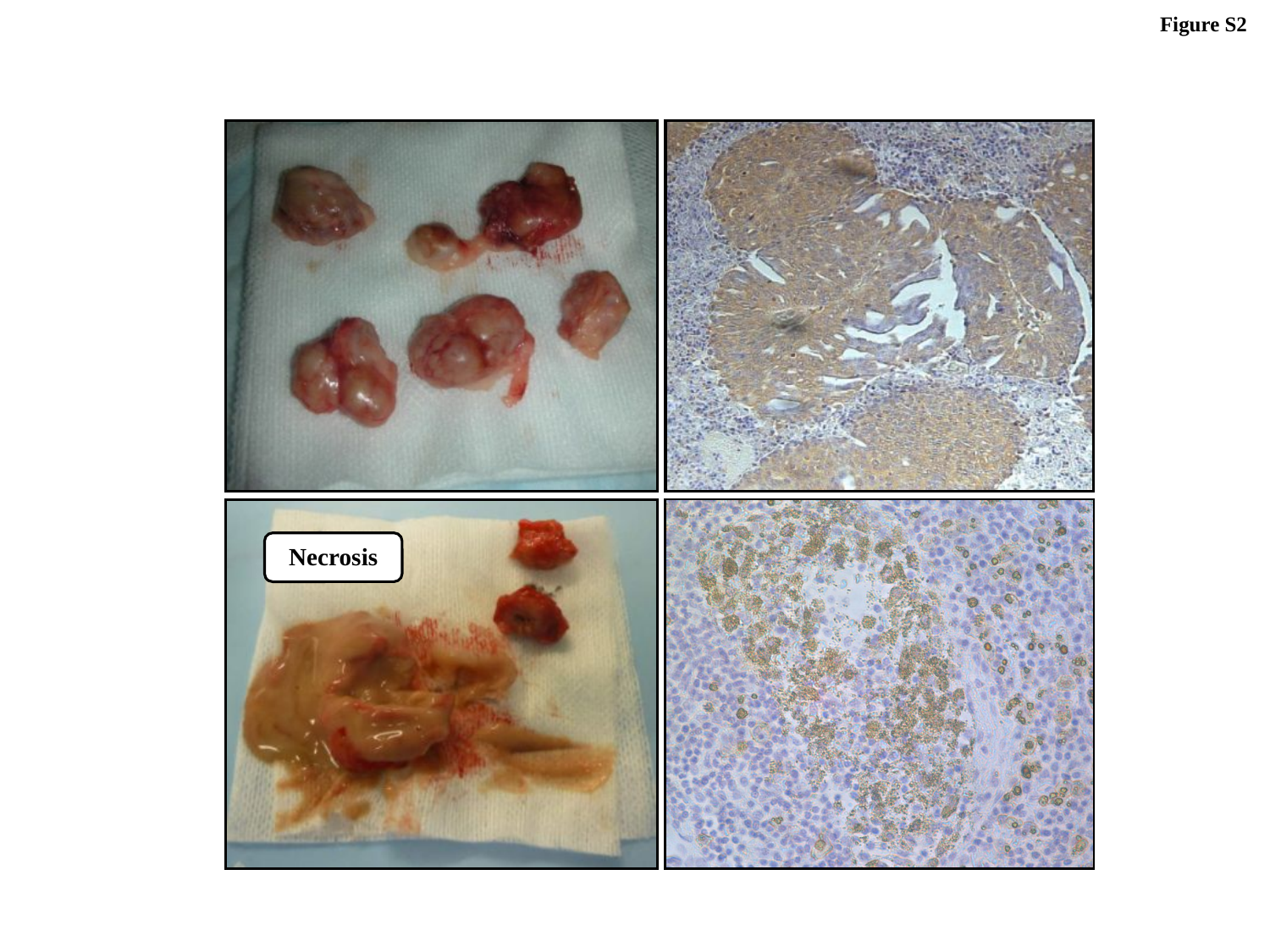

Figure S2
Necrosis

Supplement: Additional file 2: Figure S2. — PSMA expression in lymph node metastases. Sacro-iliac LNs of varying size harvested at necropsy (top left), with largest ones being often necrotic (lower left). IHC with 17G1 confirmed PSMA expression in DPC-1 cells forming LN metastases (top right) and remaining debris in necrotic ones (lower right) (×40). [file 13550_2015_155_MOESM2_ESM.pptx]
